# Suicidal behavior and Autism Spectrum Disorder, what are the risk factors? – Case Report

**DOI:** 10.1192/j.eurpsy.2023.2015

**Published:** 2023-07-19

**Authors:** C. Bayam, M. Tomé, C. Pedro, F. Cordeiro, M. J. Piçarra, L. Vale

**Affiliations:** 1 Pedopsiquiatria; 2 Psiquiatria, Centro Hospitalar e Universitário de Coimbra, Coimbra, Portugal

## Abstract

**Introduction:**

Autism is a neurodevelopmental disorder characterized by deficits in the ability to initiate and maintain social interaction, as well as a set of restricted and inflexible behavior patterns and interests. Individuals with Autism Spectrum Disorder (ASD) are at increased risk of suicidal behavior, including suicidal ideation, suicide attempts and death by suicide, as compared to the general population. Among the underlying causes, the co-occurrence of other psychiatric disorders, such as depression and anxiety, is common and can contribute to the reduction of the quality of life, as well as a worse prognosis of the disease.

**Objectives:**

Case report and brief review of risk factors associated with suicidal behavior in individuals with ASD.

**Methods:**

Review of the patients clinical file; Brief non-sistematic literature review of articles indexed to Pubmed with the key words: “Autism Spectrum Disorder”, “Suicide”, ”Suicidal behaviour”, ”Mood disorder”.

**Results:**

J., 18 years old, male, with ASD, the best student at school, with above-average results since childhood. Two years ago he showed a non-reciprocal love interest. Since then, he has had multiple visits to the emergency department and successive hospitalizations, mostly because of mood and behaviour alterations, with suicidal ideation. After 1 month with depressive and anxious symptoms, he ended up making a suicide attempt through voluntary intoxication by prescribed medication. He was taken to the emergency room. Examination of mental status highlighted depressed mood, elevated anxiety levels, hypoprosody, and active suicidal ideation. Blood tests and CE-CT scan without changes. He was admitted in the psychiatry ward and treated with fluvoxamine, risperidone and lorazepam. He showed a good evolution of the psychopathological condition. Discharged at day 44, he was referred to a psychiatric and psychological outpatient clinics.

**Conclusions:**

Mood disorders have a significant impact on the well-being of individuals with ASD, contributing to a worse quality of life and higher suicide mortality. Cognition has been associated with different levels of death by suicide, and individuals with ASD without intellectual disability, such as this patient, are at increased risk of suicide, which may be due to a greater awareness of their own difficulties. The role of genetics has been a subject of interest. The overlap of genes strongly associated with suicidal behavior and ASD has been described. However, there is still need of large scale genetic studies, for a better understanding of the genetic mechanisms involved in this association. The identification of vulnerable individuals and early initiation of preventive and therapeutic strategies is essential to improve the prognosis of ASD.

**Disclosure of Interest:**

None Declared

